# Low voltage areas, atrial natriuretic peptides and fibrosis: Challenges and controversies

**DOI:** 10.1002/clc.23497

**Published:** 2020-11-12

**Authors:** Mariana Floria, Smaranda Radu, Aurelian Corneliu Moraru, Daniela Maria Tanase

**Affiliations:** ^1^ Grigore T. Popa University of Medicine and Pharmacy Iasi Romania; ^2^ Dr. Iacob Czihac Military Emergency Clinical Hospital Iasi Romania; ^3^ Romanian Academy Iasi Romania; ^4^ Sf. Spiridon Emergency Hospital Iasi Romania

To the Editor,

We read with great interest the excellent article written by Seewöster et al.^1^ In atrial fibrillation (AF), left atrial (LA) fibrosis and dysfunction, biomarkers as atrial natriuretic peptide (ANP), delayed enhancement areas and low voltage areas (LVAs) are related to atrial cardiomyopathy and atrial failure.^2^, ^3^ Indeed, NT‐proANP could be a missing link in LVAs prediction,^1^ but there are some issues.

LVAs detected by electroanatomical mapping are a surrogate for LA fibrosis, best assessed by late gadolinium enhancement.^3^ The 0.5 mV threshold was arbitrarily chosen for LVAs. Despite this, fractioned electrograms seem to be exactly in these areas. There has been no reported correlation between LVAs and histological specimens. No consensus is available between different techniques and catheters used for LVAs detection. In addition, there are several technical challenges in determining LVAs, including electrode spacing, their positioning in relation to wavefront direction, and its contact with the LA endocardium.^3^ Difficult anatomical regions might result in undetectable LVAs caused by inappropriate contact. Detection during AF increases motion artifacts and raises difficulties by the varying directions of the wavefronts in relation to electrode positioning.^3^ However, it seems that LVAs are an independent predictor of recurrence, whether the LA is mapped in AF or sinus rhythm.^4^ In addition, delayed enhancement areas seem to be more extensive than LVAs.^5^ Both techniques lack agreement in protocols, which may lead to heterogeneities, and increased difficulties in comparing the two.^3^ Therefore, it is difficult to use LVAs as outcome. However, if LVAs correlates with NT‐proANP, why not also with LA volume?

We know that LVAs correlate with NT‐proANP and several studies have shown that they both correlate in various degrees with LA fibrosis. However, when it comes to LA volume, its relation to LA fibrosis is rather unpredictable as we can have patients with intensely fibrotic but of normal LA volume. It is exactly for this reason that the correlation with NT‐proANP might be inconsistent across studies. Moreover, the more fibrosis there is, the less LA myocardium to secrete NT‐proANP will be. Initially, increasing LA fibrosis as a form of structural remodeling correlates with low voltage areas and increased NT‐proANP levels determined by atrial stretch. LA volume is a tardive phenotypic manifestation of atrial cardiomyopathy, inconstantly correlating with NT‐proANP levels and fibrosis levels (Figure 1).

**FIGURE 1:**
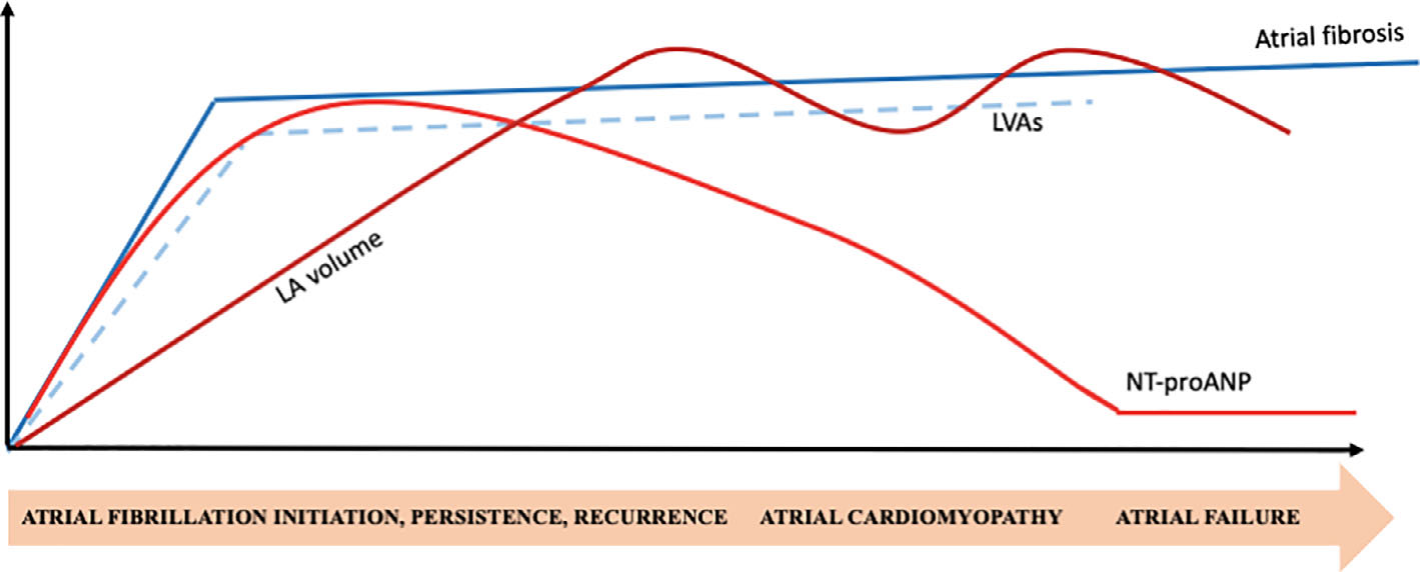
The evolution of atrial cardiomyopathy in atrial fibrillation patients and the complex relationship between LVAs, LA volume, NT‐proANP, and fibrosis. LA, left atrial; LVAs, low voltage areas; NT‐proANP, NT‐pro atrial natriuretic peptide

## Data Availability

Data sharing is not applicable to this article as no new data were created or analyzed in this study.
